# Increased P Wave Dispersion in Elite Athletes

**Published:** 2011-05-01

**Authors:** Raimundo Carmona Puerta, Ebrey Leon Aliz, Magda A Rabassa Lopez-Calleja, Ramiro Ramos Ramirez, Gustavo Padron Pena

**Affiliations:** 1 Department of Clinical Cardiac Electrophysioloy and Pacing, Cardiocenter "Ernesto Che Guevara", Santa Clara, CUBA; 2 Department of Cardiology, "Celestino Hernadez Robau" Hospital, Santa Clara, CUBA; 3 Sport Medical Center of Villa Clara, CUBA

**Keywords:** P wave duration, P wave dispersion, atrial fibrillation, athletes, healthy individuals

## Abstract

**Background:**

Few studies have been performed on P wave indices in athletes. The aim of this study was to determine the behaviour of maximum P wave duration (Pmax), minimum P wave duration (Pmin) and P wave dispersion (PWD) in young high performance athletes, as well as the relationship of PWD with training history, heart rate (HR) and echocardiographic parameters.

**Methods:**

We performed a cross-sectional observational study in 38 athletes of high performance in sports: water polo, distance running and weight lifting compared with 34 sedentary controls.

**Results:**

The average age in both groups was 20.6 years. Note that PWD was increased in athletes (57 ± 14 ms vs. 40 ± 12 ms, p <0.001) while Pmin was significantly lower (57 ± 13 ms vs. 72 ± 13 ms, p <0.001), and there was no difference when comparing Pmax (114 ± 9 ms vs. 117 ± 14 ms, p> 0.05). The correlation between the duration of training (r = 0.511) and resting HR (r = 0.461) with PWD was significant (p <0.01).

**Conclusions:**

PWD is increased in young athletes of high performance and was positively correlated with duration of training and baseline HR. The increase in PWD was secondary to a significant decrease in Pmin.

## Introduction

AF is known to be more prevalent in trained athletes compared with sedentary controls [1-5]. A study by the group of Cardiovascular Disease from the Clinical Hospital of Barcelona suggests that high-intensity exercise performed for many years may predispose to AF. The study was based on the analysis of 1160 patients over a period of two years and found a 6% prevalence of AF. In 32 of these patients (63% of those with AF) it was described as a common feature of high-intensity sport practiced for years at a young age; the incidence of AF was above the incidence observed in the general population [6].

Conduction abnormalities occurring in patients with AF can be detected by non invasive methods through the determination of P wave dispersion (PWD) in the standard 12-lead electrocardiogram (ECG) where a discontinuous and prolonged spread of atrial depolarization can be identified. The sympathetic nervous system has also been observed to influence PWD [7-9].

The P wave indices of maximum duration and dispersion have received increasing attention and have been examined in a broad range of clinical settings, especially in relation to AF [7,10]. PWD has not been widely studied in athletes, so the first papers should focus on establishing whether this parameter is modified in high performance sports.

Therefore, the aim of this study was to determine the behaviour of Pmax, Pmin and PWD in young athletes of high performance and correlate PWD with duration of training, heart rate (HR) and echocardiographic parameters.

## Materials and Methods

### Type of study

Observational analytic cross-sectional study.

### Population

A total of 38 high performance athletes from three sports: female water polo, distance running and weight lifting training at the Institution of Bodybuilding of Villa Clara were compared with 34 healthy sedentary control subjects.

### Variables studied

Age, duration of training, Pmin, Pmax, PWD, HR, left atrial size, interventricular septum thickness in diastole (ISTd), left ventricular posterior wall in diastole (LVPWd), left ventricular internal dimension in diastole (LVIDd) and h/r index.

### Definition of variables

#### PWD

Difference between maximum and minimum P wave duration in a 12-lead ECG, expressed in ms.

#### RR interval

Time between R waves, taken by the average of 3 consecutive R waves, expressed in ms

#### Left atrial size

Refers to the anteroposterior transverse diameter measured using a parasternal long axis view in ventricular systole in the echocardiogram.

#### h/r  index

Has been used to assess the metabolic rate (aerobic-anaerobic) in which the heart is working; a value below 0.32 indicates an aerobic state while a  value above 0.36 indicates an anaerobic state, [11]  determined by the ISTd  + LVPWd / LVIDd formula.

### Techniques and procedures

For the recording of the ECG we used a Nihon Kodhen Cardio fax electrocardiograph. All ECGs were digitized, allowing to amplify the image and perform a more accurate measurement. The measurements were done manually using digital calipers.

Echocardiographic parameters were obtained by an equipment Aloka 4000 Japanese technology using M mode as recommended by the American Society of Echocardiography [12]. The ECGs and echocardiograms were performed between February and April 2007. The study was approved by the instutional review board.

### Statistical analysis

The data were processed using the statistical package for social sciences (SPSS) and a  simple linear correlation was applied (Pearson). The sample means were compared by student T test. The significance levels used were: p> 0.05 (not statistically significant), p <0.05, p <0.01 and p <0.001 representing increasing levels of statistical significance.

## Results

Table 1 shows that age prevalence of both sexes was similar in both groups (athletes: 20.6 ± 5.3 years vs. controls 20.6 ± 4.9 years, p = 0.992). The duration of training was 9.9 ± 6.3 years.

Table 2 displays ECG variables showing that PWD was significantly increased in athletes: 57 ± 14 ms vs. 40 ± 12 ms, p <0.001. Pmin was significantly lower in the athletes group compared with controls (57 ± 13 ms vs. 72 ± 13 ms, p <0.001) with no significant differences when comparing Pmax (114 ± 9 ms vs. 117 ± 14 ms, p > 0.05). 

Athletes show significant lower values of HR compared with sedentary controls (70 ± 12 beats/min vs. 77 ± 11 beats/min, p <0.05)

**Figure 1:**
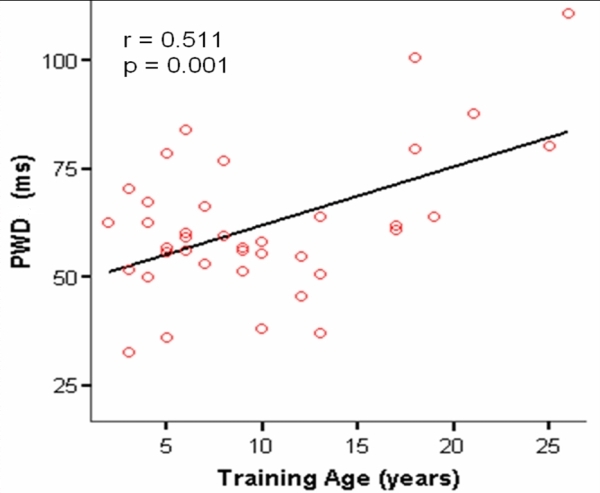
Relationship between training history and PWD in the athletes.

In Figure 1 we observe a significant correlation (r = 0.511, p <0.01) between increase in PWD and duration of training.

**Figure 2:**
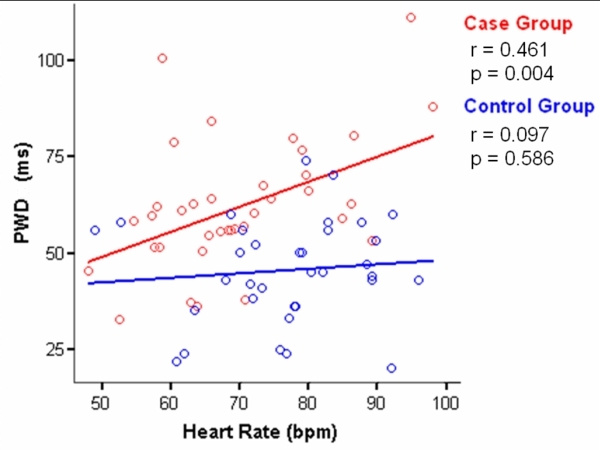
Relationship between heart rate and PWD.

Figure 2 displays the correlation between HR and PWD. A significant positive correlation between these variables was observed in the group of athletes (r = 0.461, p <0.01) whereas the control group showed a weak positive correlation (r = 0.097) which was  not statistically significant (p> 0.05).

Linear regression coefficient was obtained by correlating the left atrial size (r = - 0.098, p = 0.558) and the h/r index (r = -0.115, p = 0.492) with the PWD noting that these parameters are weakly and negatively correlated with PWD.

By applying multiple correlation to assess the combined effects of the age of athletes, duration of training and HR on the PWD, the latter shows a positive and strong correlation (r = 0.67) proved to be highly significant (p <0.001).

## Discussion

All patients studied were screened regularly because of their top-level athlete status, and did not show any detectable cardiac dysfunction which may have influenced our results. Therefore, the data obtained from these athletes reflect the influence of systematic physical exercise on atrial electrophysiology.

A recent meta-analysis based on the studies of six case controls collected 665 athletes with a mean age of 51 ± 9 years, showed that the risk of AF is higher in athletes compared with non-athletes (odds ratio 5.29 [3.57- 7.85, 95 CI]) [5]. Molina et al. observed in their cohort study that the incidence of lone AF is higher in marathon runners than in sedentary general population [2] and noted that current sport practice is associated with lone AF if lifetime sports practice is more than 1500 hours [3].  Hoogsteen et al. in their follow-up study for 9 years in endurance sports practitioners with AF diagnosis found that up to 26% of these athletes may be asymptomatic and that the arrhythmia tends to remain in the paroxysmal form [13]. They used circumferential pulmonary vein ablation to treat AF secondary to endurance sports with similar results to AF of other origin [14]. Despite the evidence previously mentioned, a recent study found no cases of AF or atrial flutter in 30 elite athletes over a 15 year follow up practicing endurance sports [15]; this discrepancy may be explained by the reduced size of the sample.

Although an increase in Pmax and PWD in athletes was associated with a likelihood to develop AF, contradictory observations have been reported. Karakaya et al. concluded in their comparative study on predictors of rhythm disorders that the athlete's heart is not associated with an increase in PWD or Pmax, and claimed that these parameters cannot be used as predictors of AF [16] in athletes with ages close to that of our athletes. However, in a recent study conducted on 27 well-trained basketball players, a significant increase was observed with respect to controls when comparing Pmax and PWD [(Pmax: 94.62 ms vs. 86.53 ms) (PWD: 49.07 ms vs. 41.15 ms)]. It should be noted that these players had ages close to those observed in our study and also included women [17]. There are very few studies reporting the behavior of P wave parameters in elite athletes. Our results satisfy the expected hypothesis that PWD of this subgroup of individuals (athletes) should be increased as been consistently demonstrated in other contexts and that sustained AF necessarily needs to have a substrate or circuit correlated with an increment in the PWD. Paradoxically, in our study, PWD was increased due to a decrease in Pmin, which has not been reported previously in athletes. We were only able to find one study with similar findings and it was performed on pregnant women compared with similar non-pregnant controls [18]. The authors did not link this result with an increased risk of AF, but Tan and Lie in an extensive review noted that the incidence and severity of supraventricular tachycardia is increased during pregnancy and that includes incessant forms of atrial tachycardia [19] which is frequently associated with AF. There are no prior reports on the mechanistic meaning of an increased PWD at the expense of a selective reduction of Pmin; we can only pose a hypothesis on this finding. It is known that as a part of the physiology of sports, functional improvements in different parameters tend to occur; a reduced Pmin means atrial areas with decreased conduction times, maybe produced by upregulation of atrial connexins, with this occurring heterogeneously. Finally, this increases PWD, which is a heterogeneity index for the propagation of the atrial impulse, by a different way than what was previously reported. This result also reveals that there is no slowing down of atrial conduction in the athletes studied. Other studies are needed attempting to replicate our findings so that they can be generalized.

We found a lower HR in the group of athletes which is part of the physiology of well-trained athletes reported previously [1,20].

Our study demonstrates that years of practicing sports (duration of training) are positively correlated with PWD. This constitutes the basis of the electrophysiological observation that established a cut-off of 1500 hours of sport practice for the appearance of an increased risk of AF [3], i.e., it takes considerable time of playing sports to display AF, this seems to be a cumulative effect.

In Figure 2 we show that HR is positively correlated with PWD, which suggests that an increased sympathetic tone leads to more atrial heterogeneity in impulse propagation with the increased vulnerability that this entails for the start of AF. It should be taken into account that resting HR values were used to make this correlation so these results should not be extrapolated to athletes performing sporting activities but rather to the athletes at rest with different levels of adrenergic tone. A clinical trial assessing the effect of exercise on P wave indices control subjects had an increase in mean dispersion (52.1 to 53.0) between rest and peak exercise [21].

In spite of being aware of the existence of adrenergic AF, no studies linking the PWD with this variety of AF in athletes have been performed. Ozdemir and colleagues showed that AF induced by exercise (in non athletes) carried out through stress tests correlates well with PWD; in addition to the observation that this parameter is higher in these patients, two weeks of treatment with different β-blockers agents reduced PWD and the recurrence of AF [22]. These results support the role of the sympathetic nervous system in the genesis of AF associated with exercise. Despite the above data, Sorokin et al, after reviewing articles published from 1960-2008 to correlate endurance athletes and AF suggested that an increased parasympathetic tone and decreased sympathetic tone are important factors to explain the increased risk of AF experienced by this population [42]. In our opinion the role of intermittent and repetitive changes in the tone of the two branches of the autonomic nervous system may be more important.

Although there was no correlation between atrial size and PWD, this relationship has been described in several studies [23-25]. We believe that having used the anteroposterior atrial diameter could have an influenced this result. This method has been recognized to be the only one that underestimates the true atrial size making it insensitive to that objective [26,27].

An inverse and very weak relationship was observed between the h/r index and PWD. PWD is larger in hearts with a more aerobic metabolic state but the change was not statistically significant. It is possible that the hearts of athletes that handle larger end diastolic ventricular volumes are associated with inhomogeneous propagation of atrial electrical impulse, but more evidence is needed to support this hypothesis.

Finally we highlighted that when one takes into account duration of training, the athlete's age and resting heart rate create an additive effect that increases PWD.

Our data suggest that athletes undergo an increase in PWD, that could be a possible mechanism for the increased incidence of AF in this group, but could also represent a physiological type of electrical remodeling without deleterious consequences.

We concluded that PWD is increased in young athletes of high performance and was positively correlated with training history and baseline HR. The increase in PWD was secondary to a significant decrease in Pmin.

A limitation of our study is the limited sample size and lack of frequent monitoring.

## Figures and Tables

**Table 1:**
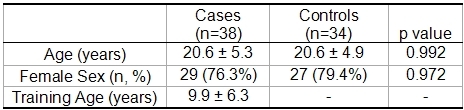
General characteristics of athletes and sedentary controls.

Values are mean ± standard deviation

**Table 2:**
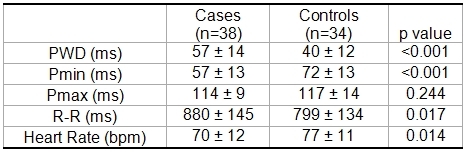
Electrocardiographic parameters of P wave obtained from the two groups

Values are mean ±standard deviation
